# Editorial: Generative AI in education

**DOI:** 10.3389/frai.2024.1532896

**Published:** 2024-12-10

**Authors:** Diego Zapata-Rivera, Ilaria Torre, Chien-Sing Lee, Antonio Sarasa-Cabezuelo, Ioana Ghergulescu, Paul Libbrecht

**Affiliations:** ^1^ETS, ETS Research Institute, Princeton, NJ, United States; ^2^Department of Informatics, Bioengineering, Robotics and Systems Engineering, University of Genoa, Genoa, Italy; ^3^Department of Computer Systems and Computing, Sunway University, Bandar Sunway, Malaysia; ^4^Department of Computer Systems and Computing, Complutense University of Madrid, Madrid, Spain; ^5^Adaptemy, Dublin, Ireland; ^6^Department of Computer Science and Data Science, IU International University of Applied Sciences, Erfurt, Germany

**Keywords:** Generative AI, personalized assessment, AI tutors, student emotions, whole learner, interactive listening tasks, AI robots, AI mentors

In the field of education, there is a growing interest in the use of Generative Artificial Intelligence (Generative AI) to reshape the educational landscape. This Research Topic investigates the transformative potential of Generative AI in various aspects of education. The papers in this edited volume shed light on the latest discoveries, new insights, novel developments, and future challenges in this rapidly advancing field.

By leveraging machine learning models, these intelligent systems extract useful insights from vast amounts of data, making them capable of delivering highly individualized content. They can analyze a learner's proficiency level, learning style, and pace, and then tailor the study material accordingly. Generative AI can adapt its content generation strategies to meet distinct preferences and learners' needs. This can increase student engagement and comprehension, highlighting its potential to transform traditional teaching methodologies.

This Research Topic also explores the use of Generative AI as part of AI tutors, capable of tailoring instructions and feedback dynamically based on each learner's progress. Acting as an ever-present mentor, Generative AI can offer learning aids beyond class hours, facilitating continuous learning and immediate doubt clarification. This can be crucial for learners encountering obstacles outside the typical school hours or during self-study periods. Anyway, to use Generative AI as a tutor, further research is needed to examine not only the accuracy of its answers but also their emotional content, as emotions play a crucial role in the learning process.

This Research Topic includes 11 papers (Original Research: six; Perspective: two; Opinion: two, and Mini-Review: one). These papers explore areas such as: (a) using Large Language Models (LLMs) to generate feedback, (b) the use and perceived usefulness of a Generative AI chatbot for schoolwork among adolescents, (c) the potential of Generative AI in supporting critical thinking and enhancing human interactions, (d) using ChatGPT to support pre-service mathematics teachers in constructing mathematical proofs, (e) opportunities and challenges of LLMs to model the “whole learner,” (f) exploring Generative AI for personalized educational assessment, (g) the use of AI-mentors in career exploration, (h) the responsible integration of AI in education, (i) the use of LLMs to automatically generate interactive listening tasks, (j) the potential of AI-enhanced robots to generate incorrect information and deceive students, and (k) the potential harm when AI-enhanced robots generate. The main contributions of these articles are described below.

*Comparing emotions in ChatGPT answers and human answers to the coding questions on stack overflow* by Fatahi et al.. This paper presents a study aimed to compare the emotional content in human and AI answers. Specifically, it examines the emotional aspects in answers from ChatGPT and humans to 2,000 questions sourced from Stack Overflow, finding that ChatGPT's answers tend to be more positive, while human responses often express anger and disgust. Additionally, human emotions exhibit a broader spectrum than ChatGPT. The authors suggest that ChatGPT shows promise as a virtual tutor for students by answering queries and fostering collaboration. However, further research is needed on the emotional aspects of responses.

*Adolescents' use and perceived usefulness of generative AI for schoolwork: exploring their relationships with executive functioning and academic achievement* by Klarin et al.. The article explores adolescents' frequency of use and perceived usefulness of generative AI chatbots for schoolwork, focusing on their relationship with executive functioning (EF) and academic achievement. Two studies were conducted with adolescents. Findings indicate that older students use Generative AI tools as more frequently. Also, students facing more EF challenges perceive Generative AI tools as more useful for completing assignments. However, no significant link was found between the use of Generative AI and academic achievement. Future work involves exploring additional Generative AI issues such as potential gender differences, implications for academic equity and the impact on adolescent cognitive development.

*Using Generative AI in education: the case for critical thinking* by Lee and Low. This opinion article makes the case for focusing the use of Generative AI in enhancing students' critical thinking and human interactions. The authors describe two case studies: (a) teaching communication skills and (b) teaching data structures and algorithms with AI chatbots. The two cases illustrate the potential use of Generative AI to enhance teaching and learning. The authors discuss the benefits of AI-based personalized feedback in improving student engagement and fostering strategic and critical use of AI tools. The article encourages the ethical and responsible use of generative AI in education with potential implications for the workforce.

*Using large language models to support pre-service teachers' mathematical reasoning—an exploratory study on ChatGPT as an instrument for creating mathematical proofs in geometry* by Dilling and Herrmann. LLMs can be a great source to extract knowledge. It thus appears natural to expect them to generate the texts of classical mathematical proofs. The authors explore how pre-service teachers employ them to produce proofs. Using the lens of instrumental genesis, their study shows a variety of usage patterns with limited knowledge about the inner workings of the models. It sketches the road to become a teacher support instrument.

*Large language models for whole-learner support: opportunities and challenges* by Mannekote et al. examines the transformative potential of LLMs in education through the development of personalized learning environments that address both cognitive and non-cognitive dimensions of learners, including motivation and socioemotional needs. The authors underscore the necessity of enhancing the interpretability of LLMs to ensure accurate learner representations, leveraging adaptive technologies for customized pedagogical support, and refining methods for authoring and evaluating educational agents. However, the article also highlights significant challenges, such as model interpretability, ethical considerations, and privacy concerns, which must be resolved.

*Opportunities and challenges of using generative AI to personalize educational assessment* by Arslan et al.. The article explores the challenges and opportunities of integrating Generative AI in supporting personalized educational assessments. The authors describe potential benefits of Generative AI personalized assessments, such as increasing learner engagement, motivation, performance, and access. Challenges include ensuring validity, reliability, and fairness. Finally, potential solutions include implementing guidelines for the ethical use of AI, aligning the purpose of the assessment with the intended use of Generative AI, and deploying human-in-the-loop approaches.

*Navigating STEM careers with AI mentors: a new IDP journey* by Chang et al.. The MyIDP, a Web-based STEM career development-mentoring platform, is the synergistic outcome of experts from diverse associations and universities (Hobin et al., 2012). Concerned with time and resource capacities, Chang et al. investigate the efficacy of a comprehensive list of prompts, when students engage with human-Google Gemini mentors. Progress/achievements in the Assessment, Career Exploration, Create Plan and Implement Plan phases, are measured by SMART goals. Findings reveal the emergence of the sequential integration and concurrent collaboration interaction models, and the importance of human mentors in refining and personalizing Gemini's more generic answers.

*Shaping integrity: why generative artificial intelligence does not have to undermine education* by Tan and Maravilla examines the role of Generative AI in promoting academic integrity. The authors argue that Generative AI can enhance learning by fostering intrinsic motivation, digital literacy, and knowledge construction. Moreover, its responsible integration can support personalized and interactive learning while upholding ethical standards. However, the paper also emphasizes the need for ethical guidelines, transparency, and thoughtful implementation to address challenges such as data privacy and algorithmic bias. Ultimately, the paper concludes that Generative AI is a tool to enrich education, preparing students for the complexities of a technologically advanced world with integrity and ethical awareness.

*A generative AI-driven interactive listening assessment task* by Runge et al.. This article discusses the development and evaluation of an interactive listening assessment task in the context of a large-scale assessment. LLMs are used to enhance automated item generation. A pilot study with 713 tasks demonstrated the feasibility of this approach, showing that AI-driven item generation can produce high-quality, diverse assessment content. The study highlights the potential of Generative AI and human-in-the loop to improve language testing by interactive assessment tasks.

*Deception detection in educational AI: challenges for Japanese middle school students in interacting with Generative AI robots* by Salem and Sumi. The authors investigate whether twenty-two Japanese middle school students can detect different types of lies (lying, paltering, pandering, and bullshitting) via an anime face in contrast to a human-like face. Analyses from ten teaching sessions indicate that there are no significant differences in learning effectiveness, and in motivation and encouragement. However, most of the students are deceived. There is also a significant difference with regards to total belief.

*Exploring the utilization and deficiencies of generative artificial intelligence in students' cognitive and emotional needs: a systematic mini-review* by Ortega-Ochoa et al. examines how Generative AI tools, like ChatGPT, address students' cognitive and emotional needs in educational contexts. The paper reviews four empirical works and notes challenges in scalability and generalizability, emphasizing the need for improved accuracy, personalization, and ethical integration of Generative AI to support meaningful and adaptive learning experiences. Furthermore, the paper highlights Generative AI's effectiveness in fostering engagement, emotional regulation, and instant feedback. However, it also identifies limitations, such as the inability to foster critical thinking, inconsistent response accuracy, and insufficient personalization to individual emotional and cognitive states.

As a whole, this Research Topic provides interesting insights regarding the use of Generative AI in education. The papers collectively explore the multifaceted roles of generative AI in education, examining its impact on emotional engagement, academic achievement, critical thinking, personalized assessment, STEM career guidance, and ethical considerations, while also addressing the challenges and opportunities it presents in shaping the future of learning and assessment. Our contribution represents an early step toward a scientific approach away from the trendy statements. The volume identifies the potential benefits and opportunities for additional work in this area. We hope you find these articles informative and help inspire new work in this active area of research. Finally, we would like to acknowledge the reviewers who participated in this Research Topic as well as Professor Rita Orji (Dalhousie University, Halifax, Canada), who served as the editor for one of the submitted manuscripts.
